# Effect of the kangaroo position on the electromyographic activity of preterm children: a follow-up study

**DOI:** 10.1186/1471-2431-13-79

**Published:** 2013-05-16

**Authors:** Kaísa Trovão Diniz, José Eulálio Cabral-Filho, Rafael Moura Miranda, Geisy Maria Souza Lima, Danilo de Almeida Vasconcelos

**Affiliations:** 1 Post Graduate Program of Institute of Integrated Medicine Prof. Fernando Figueira (IMIP), Rua dos Coelhos, 300, Boa Vista, Recife, PE, CEP 500070-550, Brazil

**Keywords:** Kangaroo-mother care method, Muscle tonus, Electromyography, Child development

## Abstract

**Background:**

One of the components of the Kangaroo Method (KM) is the adoption of the Kangaroo Position. The skin-to-skin contact and the vertical position the child adopts when in this position may provide sensorial, vestibular and postural stimuli for the newborn. The Kangaroo Position may encourage vestibular stimuli and a flexed posture of the limbs, suggesting the hypothesis that the Kangaroo Position may have an impact on flexor muscle tone. The effect of these stimuli on the motor features of the newborn has not been the subject of much investigation. No study has yet been conducted to determine whether the Kangaroo Position may progressively increase electromyographic activity or whether this increase persists until term-equivalent age. The aim of this study was to evaluate the effect of the Kangaroo Position on the electromyographic activity of preterm children.

**Method:**

A follow-up study was carried out between July and November 2011 at the Instituto de Medicina Integral Prof. Fernando Figueira (IMIP), Recife-Brazil, using a sample of 30 preterm children. Surface Eletromyography (SEMG) was used to investigate the muscle activity of biceps brachii. The electromyographic readings were taken immediately before (0 h) and after 24 h, 48 h, 72 h, 96 h of application of the Kangaroo Position as well as at the term equivalent age in each baby. Electromyographic activity was analyzed using the Root Mean Square (RMS) and the mean values of the times were analyzed by way of analysis of variance for repeated measures and the Tukey test.

**Results:**

Electromyographic activity of the biceps brachii varied and increased over the whole 96h period (RMS:0 h = 36.5 and 96 h = 52.9) (F(5.174) = 27.56; p < 0.001) and remained constant thereafter (RMS: term-equivalent age = 54.2). The correlations between the corrected age and the values for electromyographic activity did not show any statistical significance.

**Conclusion:**

The Kangaroo Position leads to a growing increase in the electromyographic activity of preterm children’s biceps brachii after up to 96 h of stimulation and this response persists until at least the 21st day after this period.

## Background

One of the most important current neonatal health care initiatives is the Kangaroo Method (KM), which is a care method intended for preterm low-weight newborns [1]. There is growing evidence that the KM brings benefits that are both physiological [2-7], such as improvement in the vital signs, oxygenation of the brain and reduction in the pain response, and behavioral [1,8,9], such as improved sleep and less crying in the preterm newborn, and also improves adherence to and duration of maternal breastfeeding [4,9,10] and mother-child bonding [1]. This method is also associated with a reduction in time spent in hospital and in mortality among preterm newborns [4,11]. One of the components of the Program is the adoption of the Kangaroo Position which is the trademark of the method. In this position, the newborn should be dressed in light clothes, lying face down vertically against the adult’s chest with limbs flexed [1]. The skin to skin contact and the vertical position the child adopts during this method may provide sensorial, vestibular and postural stimuli for the newborn.

The effect of these stimuli on motor features of the newborn has not been the subject of much investigation [12-14]. In view of this and, given that preterm newborns usually exhibit muscular hypotonia with posturing that is manifested mostly in extension, rather than in flexion; [1,15]; that the vestibular system helps to maintain muscle tone [16]; and also that the Kangaroo Position may encourage vestibular stimuli and a flexed posture of the limbs [1], there is reason to suggest the hypothesis that the Kangaroo Position may have an impact on the flexor muscle tone of preterm newborns.

A previous study conducted in the city of Recife, Brazil using Surface Eletromyography (SEMG) found an increase in the electromyographic activity of flexor muscles in babies adopting the Kangaroo Position for 24 h and that this effect persists for 24 h thereafter [13]. However, no study has yet been conducted to determine whether the Kangaroo Position may progressively increase electromyographic activity or whether this increase persists until term-equivalent age.

The aim of this study was thus to determine the electromyographic profile of preterm newborns at different stages during adoption of the Kangaroo Position and term equivalent age.

## Methods

### Participants

A follow-up type study was carried out between July and November 2011 at the Instituto de Medicina Integral Prof. Fernando Figueira (IMIP), in Recife, Brazil with 30 preterm newborns hospitalized at the Institute’s Kangaroo Unit.

The IMIP’s Kangaroo Unit covers a surface-area of 600 m^2^ and has a ward with 22 beds for clinically stable preterm newborns (with a respiratory frequency of between 30–60 inspirations per minute, a heart rate of between 120–160 beats per minute, peripheric oxygen saturation of over 89%, absence of signs of respiratory distress, absence of cyanosis or pallor and pain.) The newborns had to tolerate food, to breathe without the use of an apparatus and to weigh more than 1,250 grams.

The Kangaroo Unit provides medical and nursing services and also speech therapy and physiotherapy. In this unit, the newborns, when referred by the medical services, are evaluated and undergo an early stimulation program.

Preterm newborns considered eligible for inclusion in the sample were those with a gestational age of 27 to 34 weeks and a corrected age of less than 35 weeks during the first electromyographic evaluation, who had not previously experienced the Kangaroo Position. The exclusion factors were: an Apgar of less than 7 in the 5th minute, a prior history of grade III or grade IV intracranial hemorrhage (diagnosed using transfontanellar ultrasound and registered in medical records), seizures, congenital infection (cytomegalovirus, rubeola, toxoplasmosis, syphilis and vertically-transmitted HIV), malformations of the Central Nervous System (hypdrocephalia and genetic syndromes) Central Nervous System infections (meningitis or encephalitis), congenital cardiopathy, trauma during delivery (damage to the brachial plexus, luxation of the hip and fractures of the pelvis) and gastro-esophageal reflux disease.

**Figure 1:**
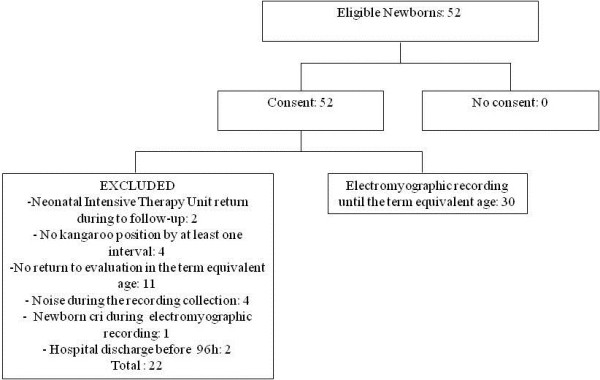
Presents a flowchart showing the number of elegible newborns.

All these inclusion and exclusion factors were assessed using data collected from the records of patients diagnosed by neonatologists from the IMIP’s Neonatal Intensive Care Unit and the Kangaroo Unit. Figure 1 presents a flowchart showing the number of elegible newborns who were excluded and the reason for their exclusion.

A convenient non-probabilistic sequential sample was obtained from the newborns. The sample comprised 30 newborns aged between 27 and 34 weeks (with a mean age of 31.21 ± 2.1) and a mean weight, in grams, of 1407.3 ± 432.1. Other information on the sample can be found in Table 1 of the Results item.

**Table 1:** Clinical and biological characteristics of the newborns

**Characteristics**	**n = 30**
Gestational Age, weeks	31.21 (2.1)
Weight at birth, grams	1407.3 (432.1)
Apgar at 5 mins of life, Md (min-max)	9 (7–10)
Corrected age on first reading (0h), weeks	34.6 (1.6)
Time elapsing between being discharged from the KU and evaluation at TEA, days	21.52 (10.9)

The mean (SD) is given for continuous variables; category variables are given in terms of absolute frequencies and percentages; and ordinal variables (Apgar) in terms of the median (min-max).

Abbreviations: TEA, Term Equivalent Age; KU, Kangaroo Unit.

The sample size was calculated using a pilot study, which confirmed the standard deviation in differences in electromyographic responses before and after adoption of the 9.3 microvolt Kangaroo Position and an estimate of the difference between the means of the two situations equal to five microvolts. With an alpha error of 0.05 and power of 0.8, the resulting sample size was 29.2 (rounded up to 30) individuals.

The project was submitted to the IMIP’s Ethics Committee for Research involving Human Beings and was approved (protocol no. 1902). The parents or guardians who agreed to participate signed a Term of Free and Informed Consent.

### Data collection

The electromyographic signal was obtained using a Miotool *400*® electromyograph (Miotec Equipamentos Biomédicos – Brazil). A system of channels and self-adhesive 4.2-mm-diameter Ag/AgCl electrodes (Meditrace® 100) were used to connect the body to the system. The electromyograph was connected to a laptop with Miographic 2.0 software (Miotec Equipamentos Biomédicos – Brazil) to process the signal. The sampling frequency was 2000 Hz. The electromyograms were magnified 2000-fold.

### Collection procedure

Readings were registered using two electrodes placed in the central portion of the muscle of the biceps brachii, between the motor point and the myotendinous junction, parallel to the muscle fibers, as recommended by the Surface Electromyography for the Non-Invasive Assessment of Muscles (SENIAM) project [17] and also unilaterally. The electrodes were adjusted to ensure that the distance between them did not exceed 20 mm. The reference electrode was always placed in the right lateral malleolus.

The electromyographic signal of the newborns was captured with the aid of a small cushion in the shape of a 30° angle wedge, called a wedge cushion. The electromyographic records were read when the newborns were in Brazelton state 4 or 5, inactive or active alert, respectively [18].

The first electromyographic reading was taken before adoption of the Kangaroo Position (0 h). Immediately after taking the first reading, the children were placed for the first time in the Kangaroo Position. The Kangaroo Position adopted was that recommended by the Neonatal Unit, in which the newborn is positioned against the adult’s chest, face down, wrapped in a strip of flexible cloth. Subsequent readings were taken after 24 h, 48 h, 72 h, 96 h of the Kangaroo Position and, finally, at term-equivalent age (40 ± 1 weeks). On each of these occasions, two readings were taken, the first with the arm at rest and the second with the arm flexed in response to the Dubowitz maneuver [19]. Each reading was carried out for a period of 30 seconds.

For the first 96 h the babies were kept in the Kangaroo Position for 8–12 hours per day. The babies were removed from the Kangaroo Position (and placed on a soft cushion) for short intervals when the mothers went to the restroom or the shower and during breastfeeding or other forms of feeding.

During data collection, the researchers asked the Kangaroo Unit not to give the newborns physiotherapy. The newborns did not, therefore, undergo any kind of early motor stimulation during data collection, except for oral stimulation by speech therapists, when necessary.

### Data processing

For signal analysis this was transformed into Root Mean Square (RMS) and normalized [20,21]. The data processing software enables RMS values to be obtained during the acquisition period. For the purpose of normalization, a figure of 100%, corresponding to the maximum peak during the Dubowitz maneuver, was taken as a point of reference and used to normalize every signal. A period of 10 s of the total electromyographic reading (30 s) was used.

### Statistical analysis

One-way analysis of variance for repeated measures was carried out to compare the means for electromyographic activity at different intervals. Tukey test for multiple comparisons was also performed to compare each two intervals of electromyographic records. To exclude the effect of the development of the child *per se* as a confounding variable, correlation was made between corrected age and the electromyographic data before adoption of the Kangaroo position (0 hrs). The alpha error for rejection of the null hypothesis was 0.05.

## Results

The mothers of the newborns had a mean age of 27.2 ±6.7 years. The clinical characteristics of the mothers and the clinical and biological characteristics of the newborns are presented in Table 1. Table 2 includes the RMS values for each point in time examined.

**Table 2:** RMS of left biceps brachii of preterm newborns placed in the Kangaroo position

**Intervals**	$\mathrm{RMS}\left(\bar{X}\pm \mathrm{DP}\right)$	**Differences between the RMS means**	
		**∆**	**%∆**
0 h	36.6 ± 9.7		
		3.9	10.65
24 h	40.5 ± 6.9		
		2.9	7.16
48 h	43.4 ± 7.5		
		3.7	8.52
72 h	47.1 ± 6.5		
		5.8	12.10
96 h	52.9 ± 8.5		
		1.3	2.45
TEA	54.2 ± 11.2		

Comparisons: Analysis of variance for repeated measurements: F(5,174) = 27.56; p < 0.001.

Tukey Test for multiple comparisons (each pair of intervals – consecutive or not): 0 h x 48 h (p = 0.03); 0h x 72 h (p < 0.001); 0h x 96 h (p < 0.001); 0h x TEA (p < 0.001); 24 h x 72 h (p = 0.005); 24 h x 96 h (p < 0.001); 24 h x TEA (p < 0.001); 48 h x 96 h (p < 0.001); 48 h x TEA (p < 0.001); 72 h x 96 h (p = 0.002); 72 h x TEA (p = 0.002).

Only statistically significant figures are presented here.

Abbreviations: RMS, Root Mean Square; TEA, Term equivalent age.

The RMS varied significantly throughout the study period: (F_(5,174)=_27.56; p < 0.001). Multiple *post hoc* comparisons (Tukey test) showed that, compared with the reading for 0h, the RMS was higher at 48 h (p = 0.03), 72 h (p < 0.001) and 96 h (p < 0.001). Moreover, there was a growing increase in RMS up to 96 h, between the following intervals: from 0 h-48 h (36.5-43.4; p = 0.03); from 24 h-72 h (40.5-47.1; p < 0.005); from 48h-96 h (43.4-52.9; p < 0.001); and from 72 h-96 h (47.1-52.9; p = 0.002).

Comparison of the RMS at term equivalent age with the other figures showed that it was significantly higher than the readings for 0 h (p < 0.001), 24 h (p < 0.001), 48 h (p < 0.001), and 72 h (p < 0.001). However, there was no significant difference compared to the reading for 96 h (p > 0.99).

**Figure 2:**
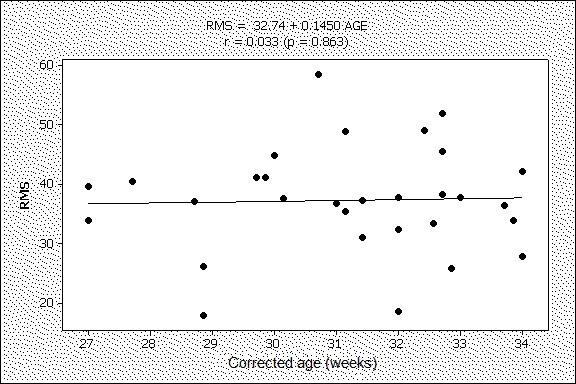
**Correlation between RMS at 0 h and corrected age of preterm newborns at the same point in time.** RMS: Root Mean Square. It can be seen that there was not significant association between RMS and corrected ages at 0 h (i.e. before adoption of the Kangaroo Position), which may indicate that electromyographic activity is not related to the growth of the newborn.

The correlation (Figure 2) between RMS (0 h) and corrected age of the preterm newborn likewise was not statistically significant (r = 0.033, p = 0.863).

## Discussion

The results of the present study show a significant increase in electromyographic activity of the bíceps brachii muscle in newborns who have been placed in the Kangroo Position. Furthermore this increase was found to be steady and constant. This finding is worthy of note, as it suggests that this position produces a functional alteration in the muscle involved in the anti-gravity posture system.

A previous study showed that early psychomotor stimulation of preterm newborns from the first to fifth corrected month of life improves motor development and that the more stimulation there is, the larger the number of motor skills acquired in the supine, prostrate and sitting positions [22]. Likewise, a meta-analysis published in 2012 shows that vestibular stimuli also improve the psychomotor development of breastfeeding preterm children at a corrected age of six and twelve months [23].

Although these other studies have examined the effect of psychomotor or sensorial stimulation on the motor development of preterm newborns, they did not specifically investigate the influence of the Kangaroo Position on this development [22-26].

Few studies have been conducted that examine the effect of the Kangaroo Position on motor development [12-14]. Barradas et al. [12] showed that preterm newborns who were placed in the Kangaroo Position adopted a posture with more flexion. Ferber & Makhoul [14] showed that, in term children, the Kangaroo Position caused an increase in flexion movements and a decrease in extensor movements compared to those not placed in this position, suggesting that the Kangaroo Position leads to an alteration in the motor system in these babies. More recently, Barradas [13] studied the influence of the Kangaroo Position on electromyographic activity in preterm newborns and found that, after 24 h of the Kangaroo Position, these newborns exhibited greater electromyographic activity in various muscle groups.

It may be that the stimulation of the kinesthetic and vestibular systems provided by the Kangaroo Position influenced the motor response observed in the aforementioned studies. The hypothesis that the vestibular and kinesthetic systems are involved in this process is backed up by the results presented in meta-analyses of interventions other than the Kangaroo Position [23,26].

Studies that showed the influence of the Kangaroo Position on the motor skills of newborns also found that this effect is persistent [13,14]. Ferber & Makhoul [14] found alterations in the motor skills of term children three hours after interruption of the Kangaroo Position and Barradas [13] has shown that the effect that the Kangaroo Position has on electromyographic activity in preterm newborns persisted for more than 24 hrs after ceasing to adopt the position. In the present study, the level of electromyographic activity at term equivalent age was no different from that recorded at 96 h, suggesting a more prolonged persistent effect.

Although we cannot rule out the possibility that the growing increase in electromyographic activity observed in the first 96 h of the Kangaroo Position may be due to the natural growth of the child, this is implausible, since there were no correlations between the ages of the newborns or their birth-weight and the level of electromyographic activity. Moreover, the results show that, even after an interval of 21 days between being discharged from hospital and the electromyographic reading at term equivalent age, the electromyographic responses at this point were similar to those found by the reading at 96 h.

Furthermore, since the newborns did not undergo any motor intervention during the data collection period, the results show that the Kangaroo position is associated with the electromyographic responses found.

One limitation of the present study may be the fact there was no comparison with a control group of newborns not placed in the Kangaroo Position, which might have revealed that the increase in RMS is not related to the Kangaroo Position but the result of the natural development/growth of the child. This limitation, however, is minimized by the lack of correlation between the age and the electromyographic activity.

Another limitation is the fact that the results cannot be generalized for newborns with a gestational age of under 27 weeks and it is not known whether the RMS would increase, were the period of adoption of the Kangaroo position shorter than that used for the present study.

The present study is, so far as we are aware, the first follow-up study of the effect of the Kangaroo Position on electromyographic activity in preterm children. Given the results found, it would be particularly interesting to investigate other muscles involved in the postural system, and to conduct a study similar to this one with term children and children who have not been placed in the Kangaroo Position.

## Conclusions

In short, the results of the present study provide further evidence that the Kangaroo Position causes an increase in the electromyographic activity of the biceps brachii in preterm newborns, increasing over 96 h and persisting until term equivalent age. Given that the electromyographic test was carried out on the muscle at rest, the increase in this myoelectric activity may suggest an increase in flexor muscle tone in the children, at least in the case of the muscle studied. It could therefore be that the Kangaroo Position provides not only physiological and behavioral benefits, as shown in other studies, but may also be associated with alterations in muscle function, improving the posture and mobility of the child adopting this position.

## Abbreviations

CNS: Central nervous system; IMIP: Instituto de Medicina Integral Prof. Fernando Figueira; KM: Kangaroo Method; PTNB: Preterm Newborn; RMS: Root Mean Square; SENIAM: Surface Electromyography for the Non-Invasive Assessment of Muscles; SEMG: Surface Electromyography.

## Competing interests

The authors declare that they have no competing interests.

## Authors’ contributions

The final submission of the manuscript was approved by all authors. KTD: Preparation of the study project, data collection and supervision thereof, statistical analysis, research articles in the database, preparation of the article. JECF: Guidance for the preparation of the study project, supervision of data collection, statistical analysis, research articles in the database; guidance and preparation of the article. RMM: Preparation of the study project, data collection and supervision thereof, data processing, statistical analysis, research articles in the database, preparation of the article. GMSL: Preparation of the study project, data collection and supervision thereof. DAV: Preparation of the study project, supervision of data collection, data processing.

## Pre-publication history

The pre-publication history for this paper can be accessed here:

http://www.biomedcentral.com/1471-2431/13/79/prepub

## References

[B1] Nyqvist KH Anderson GC Bergman N Cattaneo A Charpak N Davanzo R Ewald U Ibe O Ludington-Hoe S Mendoza S Pallás-Allonso C Ruiz Peláez JG Sizun J Widström AM Towards universal kangaroo mother care: recommendations and report from the first European conference and seventh international workshop on kangaroo mother care Acta Paediatr 2010 99 6 820 826 10.1111/j.1651-2227.2010.01787.x 20219044

[B2] Almeida CM Almeida AFN Forti EMP Effects of kangaroo mother care on the vital signs of low-weight preterm newborns Rev Bras Fisioter 2007 11 1 1 5 10.1590/S1413-35552007000100002

[B3] Begum EA Bonno M Ohtani N Yamashita S Tanaka S Yamamoto H Kawai M Komada Y Cerebral oxygenation responses during kangaroo care in low birth weight infants BMC Pediatr 2008 8 51 10.1186/1471-2431-8-51 18990243 PMC2585079

[B4] Conde-Agudelo A Belizán JM Diaz-Rossello J Kangaroo mother care to reduce morbidity and mortality in lown birtthweight infants Cochrane Database Syst Rev 2012 7 CD002771 10.1002/14651858.CD002771 11034759

[B5] Cong X Cusson RM Walsh S Hussain N Ludington-Hoe SM Zhang D Effects of skin-to-skin contact on autonomic pain responses in preterm infants J Pain 2012 13 7 636 645 10.1016/j.jpain.2012.02.008 22595172

[B6] Johnston CC Filion F Campbell-Yeo M Goulet C Bell L McNaughton K Byron J Aita M Finley GA Walker CD Kangaroo mother care diminishes pain from heel lance in very preterm neonates: a crossover trial BMC Pediatr 2008 8 13 10.1186/1471-2431-8-13 18435837 PMC2383886

[B7] Miltersteiner AR Miltersteiner DR Rech VV Mole LD Physiological responses of the kangaroo mother position in low birth weight, spontaneous ventilating premature babies Rev Bras Saude Matern Infant 2003 3 4 447 455 10.1590/S1519-38292003000400009

[B8] Feldman R Weller A Sirota L Eidelman AI Skin-to-skin contact (kangaroo care) promotes self-regulation in premature infants: sleep-wake cyclicity, arousal modulation, and sustained exploration Dev Psychol 2002 38 2 194 207 11881756 10.1037//0012-1649.38.2.194

[B9] Moore ER Anderson GC Bergman N Dowswell T Early skin-to-skin contact for mothers and their healthy newborn infants Cochrane Database Syst Rev 2012 7 CD003519 10.1002/14651858.CD003519.pub3 PMC3979156 22592691

[B10] Lamy Filho F Silva AAM Lamy ZC Gomes MASM Moreira MEL Evaluation of the neonatal outcomes of the kangaroo mother method in Brazil J Pediatr (Rio J) 2008 84 5 428 435 10.2223/JPED.1821 18923784

[B11] Lawn JE Mwansa-Kambafwile J Horta BL Barros FC Cousens S Kangaroo mother care to prevent neonatal deaths due to preterm birth complications Int J Epidemiol 2010 39 Suppl 1 i144 i154 20348117 10.1093/ije/dyq031 PMC2845870

[B12] Barradas J Fosceca A Guimarães CLN Lima GMS Relationship between positioningof premature infants in kangaroo mother care and early neuromotor development J Pediatr (Rio J) 2006 82 6 475 480 17171208 10.2223/JPED.1565

[B13] Barradas J Kangaroo position effect on the flexor muscle tone of newborn preterm 2010 Fernando Figueira: Dissertation. Post Graduate Department the Institute of Integrated Medicine Prof

[B14] Ferber SG Makhoul IR The effect of skin-to-skin contact (kangaroo care) shortly after birth on the neurobehavioral responses of the term newborn: a randomized. Controlled Trial Pediatrics 2004 113 4 858 865 10.1542/peds.113.4.858 15060238

[B15] Bracewell M Marlow N Patterns of motor disability in very preterm children Ment Retard Dev Disabil Res Rev 2002 8 4 241 248 10.1002/mrdd.10049 12454900

[B16] Strobel H Electromyographic studies on the influence of vestibular apparatus on the tonic activity of human striated muscles Arch Klin Exp Ohren Nasen Kehlkopfheilkd 1971 198 2 187 205 10.1007/BF00312232 5314907

[B17] Hermens JH Freriks B Klug CD Rau G Development of recommendations for SEMG sensors and sensor placement procedures J Electromyogr Kinesiol 2000 10 5 361 374 10.1016/S1050-6411(00)00027-4 11018445

[B18] Als H Tronick E Lester BM Braselton TB The Braselton neonatal behavioral assessment scale (BNBAS) J Abnorm Child Psychol 1977 5 3 215 229 10.1007/BF00913693 903518

[B19] Dubowitz L Ricciw D Mercuri E The Dubowitz neurological examination of the full-term newborn Ment Retard Dev Disabil Res Rev 2005 11 1 52 60 10.1002/mrdd.20048 15856443

[B20] Bolgla LA Uhl TL Reliability of electromyographic normalization methods for evaluating the hip musculature J Electromyogr Kinesiol 2007 17 1 102 111 10.1016/j.jelekin.2005.11.007 16423539

[B21] Lehman GJ McGill SM The importance of normalization in the interpretation of surface electromyography: a proof of principle J Manipulative Physiol Ther 1999 22 7 444 446 10.1016/S0161-4754(99)70032-1 10519560

[B22] Field TM Schanberg SM Scafidi F Bauer CR Vega-Lahr N Garcia R Nystrom J Kuhn C Tactile/kinesthetic stimulation effects on preterm neonates Pediatrics 1986 77 5 654 658 3754633

[B23] Symington AJ Pinelli J Developmental care for promoting development and preventing morbidity in preterm infants Cochrane Database Syst Rev 2012 7 CD001814 10.1002/14651858.CD001814 11034730

[B24] Formiga CKMR Pedrazzani ES Tudella E Motor development of preterm infants submitted to an early physiotherapist intervention program Rev Bras Fisioter 2004 8 3 239 245

[B25] Maguire CM Walther FJ Van Zwieten PHT Cessie SL Wit JM Veen S Follow-up outcomes at 1 and 2 years of infants born less than 32 weeks after newborn individualized developmental care and assessment program Pediatrics 2009 123 4 1081 1087 10.1542/peds.2008-1950 19336365

[B26] Vickers A Ohlsson A Lacy J Horsley A Massage for promoting growth and development of preterm and/or low birth-weight infants Cochrane Database Syst Rev 2012 7 CD000390

